# Astaxanthin Attenuates Hypertensive Vascular Remodeling by Protecting Vascular Smooth Muscle Cells from Oxidative Stress-Induced Mitochondrial Dysfunction

**DOI:** 10.1155/2020/4629189

**Published:** 2020-04-14

**Authors:** Yuqiong Chen, Su Li, Yuxuan Guo, Hang Yu, Yandong Bao, Xin Xin, Huimin Yang, Xinzhu Ni, Nan Wu, Dalin Jia

**Affiliations:** ^1^Department of Cardiology, The First Affiliated Hospital of China Medical University, Shenyang, Liaoning, China; ^2^Department of Cardiology, Shanghai Institute of Cardiovascular Diseases, Zhongshan Hospital, Fudan University, Shanghai, China; ^3^The Central Laboratory, The First Affiliated Hospital of China Medical University, Shenyang, Liaoning, China

## Abstract

Oxidative stress aggravates mitochondrial injuries and accelerates the proliferation of vascular smooth muscle cells (VSMCs), which are important mechanisms contributing to vascular remodeling in hypertension. We put forward the hypothesis that Astaxanthin (ATX), known to possess strong features of antioxidant, could attenuate vascular remodeling by inhibiting VSMC proliferation and improving mitochondrial function. The potential effects of ATX were tested on spontaneously hypertensive rats (SHRs) and cultured VSMCs that injured by angiotensin II (Ang II). The results showed that ATX lowered blood pressure, reduced aortic wall thickness and fibrosis, and decreased the level of reactive oxygen species (ROS) and H_2_O_2_ in tunica media. Moreover, ATX decreased the expression of proliferating cell nuclear antigen (PCNA) and ki67 in aortic VSMCs. *In vitro*, ATX mitigated VSMC proliferation and migration, decreased the level of cellular ROS, and balanced the activities of ROS-related enzymes including NADPH oxidase, xanthine oxidase, and superoxide dismutase (SOD). Besides, ATX mitigated Ca^2+^ overload, the overproduction of mitochondrial ROS (mtROS), mitochondrial dysfunction, mitochondrial fission, and Drp1 phosphorylation at Ser616. In addition, ATX enhanced mitophagy and mitochondrial biosynthesis by increasing the expression of PINK, parkin, mtDNA, mitochondrial transcription factor A (Tfam), and PGC-1*α*. The present study indicated that ATX could efficiently treat vascular remodeling through restraining VSMC proliferation and restoring mitochondrial function. Inhibiting mitochondrial fission by decreasing the phosphorylation of Drp1 and stimulating mitochondrial autophagy and biosynthesis via increasing the expression of PINK, parkin, Tfam, and PGC-1*α* may be part of its underlying mechanisms.

## 1. Introduction

Vascular smooth muscle cells (VSMCs) are the main cellular components in the arteries and possess functions to maintain the structural and physiological integrities of vessels. The principal functions of VSMCs are contracting and controlling blood pressure. However, these functions are affected in hypertension due to their phenotypic changes [1]. Unlike many other mature cells, VSMCs retain a high degree of plasticity, they can transform from a contractile state to a highly synthetic phenotype [2]. In hypertension, VSMCs become highly proliferative and produce high level of extracellular matrix components, including collagen and elastin, all of which contribute to vascular remodeling and stiffness [3]. It has been well established that these changes are primarily influenced by hemodynamic, ROS, and vasoactive substances including Ang II and aldosterone (ALD) [4, 5]. In addition, several studies suggested that NADPH oxidase-4 (Nox4) is a critical marker for VSMC differentiation due to NOX4-generated superoxide radicals are extensively involved in VSMC hypertrophy, proliferation, migration, and inflammation [6, 7].

Mitochondria are both the target and the source of ROS. Overproduced oxidant radicals impair mitochondria and lead to mitochondrial dysfunction. In order to maintain homeostasis, damaged mitochondria are eliminated through quality control measures via mitochondrial dynamics, mitophagy, and mitochondrial biogenesis [8]. In response to oxidative stress, mitochondria in proliferative VSMCs shift from fusion to fission, becoming small and disorganized [9]. What is more, Drp1, the primary regulator of fission, was found to stimulate VSMC proliferation in many disease states [9, 10]. Mitophagy and mitochondrial biogenesis are effective ways to selectively eliminate damaged mitochondria or generate new mitochondria under environmental pressures. Pharmacological activation of mitophagy and mitochondrial biogenesis can restore mitochondrial dysfunction, enhance oxidative metabolism, and improve cardiovascular diseases [11].

Astaxanthin (ATX) belongs to the xanthophyll group and has a great reputation for its outstanding antioxidant capacity to neutralize free radicals and balance prooxidant and antioxidant [12]. Currently, accumulating evidences also prove that ATX has multiple beneficial effects, such as anti-inflammation, antiapoptosis, and antiobesity activities [13]. Importantly, ATX has been suggested to reduce blood pressure and prevent vascular remodeling in SHRs [14–16]. However, the underlying mechanisms are still not fully understood. Recently, several studies have attempted to explore the protective effects of ATX on mitochondria in oxidative stress-associated diseases such as aging, fatty livers, or metabolic disorders, whereas its potential benefits on mitochondria in hypertension remain unclear [17–19]. Therefore, we aimed to investigate the potential effects of ATX on hypertensive vascular remodeling and explore the potential mechanisms involved.

## 2. Materials and Methods

### 2.1. Animals and Treatments

16 male SHRs and 16 male Wistar-Kyoto rats (WKYs), at 5 weeks of age and 140-165 g of weight, were purchased from Beijing Vital River Laboratory Animal Technology Co., Ltd. (China). All rats were fed with water and ordinary forage. At 6 weeks of age, the systolic blood pressure (SBP) and diastolic blood pressure (DBP) of SHRs were significantly higher than that of WKY rats. Then, the animals were randomly assigned to four groups: WKY group (*n* = 8), ATX-treated WKY group (*n* = 8), SHR group (*n* = 8), and ATX-treated SHR group (*n* = 8). In ATX-treated groups, 200 mg/kg of ATX was administered by intragastric injection once a day for 11 weeks according to a previous study [16]. The untreated groups were gavaged with equivalent normal saline. Animal experiments were approved by the China Medical University Institutional Ethics Committee and followed the Guide for the Care and Use of Laboratory Animal (the US National Institutes of Health publication, Doc. 2011-11490).

### 2.2. Blood Pressure Measurement and Sample Collection

SBP and DBP were monitored every week by tail-cuff method. Every measure was repeated 3 times to calculate the average blood pressure. On expiration of the experiment, all rats were executed by carbon dioxide suffocation to isolate the thoracic aorta. Every aorta was divided evenly into three parts. One part was rapidly immersed in ice-cold Krebs-Hensleit's solution and cut transversely at 3 mm to prepare vascular rings for the detection of contractility. Half of the above samples were tenderly rubbed by stainless steel wire to wipe off the endothelium mechanically. Another part was fixed by 10% formaldehyde and embedded in paraffin or Tissue-Tek® O.C.T compound to make sections. The last part was stored at -80°C for further studies.

### 2.3. Serological Experiment

Blood samples were collected from the tail vein at the end of the study. Serums were separated by centrifugation at 4°C, 1200 g for 15 min. SOD activity, glutathione peroxidase (GSH-PX) activity, the contents of MDA, glutathione (GSH), Ang II, ALD, and renin in the serum were detected by colorimetric reagent kits (Nanjing Jiancheng Bioengineering Institute, China).

### 2.4. Vascular Function Assessment

The aortic rings were connected to the Wire Myograph System (DMT620M, Denmark). After equilibrated in oxygenated Krebs-Hensleit's solution at 37°C for 45 min, the aortic rings were stretched to an ideal resting tension (1.5 g). Contractile response to 120 mM KCl was seated as a standard value for contractility, and all the following values of vasoconstrictions were standardized by this value. Afterward, the contractility of aortic ring response to different concentrations of phenylephrine (0.1 nM–0.3 mM) was detected. As for the Ca^2+^-induced vasoconstriction, the aortic rings were immersed in Ca^2+^-free Krebs-Hensleit's solution with 60 mM KCl for 45 min. Then, the contractility of aortic ring response to different concentrations of CaCl_2_ (0.1 mM–10 mM) was measured.

### 2.5. Histological Staining Analysis

Tissue sections were stained by H&E staining and observed under the Leica DM3000 microscope (Leica, Germany) to measure aortic wall thickness (WT), wall cross-sectional area (WCSA), lumen diameter (ID), and wall/lumen ratio. Fibers and collagens were stained by Masson's trichrome staining and Sirius-red staining. Quantitative analyses were performed by ImageJ.

After deparaffinage, rehydration, and permeabilization in 1‰ Triton X-100, double-immunofluorescence staining was performed on paraffin sections. The VSMCs in the aorta were labeled by anti-*α*-SMA antibody (Sigma Aldrich, USA). The ratio of NOX4-positive VSMCs and the expression of SOD2 in aortic VSMCs were marked with anti-NOX4 antibody and anti-SOD2 antibody (Sigma Aldrich, USA), respectively. Proliferative VSMCs were labeled with anti-PCNA antibody (Sigma Aldrich, USA) and Ki67 antibody (Sigma Aldrich, USA), respectively.

### 2.6. ROS Detection in the Aorta

ROS generation in tunica media was detected by Lucigenin chemiluminescence reagent kit (Genmed Scientifics, USA). After detaching the intima and adventitia, the tunica media was grinded in wash buffer. Then, the wash buffer was centrifuged to remove tissue fragments. After the lucigenin was added to the supernatant, the relative light unit (RLU) was detected by a chemiluminescence apparatus. The H_2_O_2_ content was detected by Amplex Red Hydrogen Peroxide/Peroxidase Assay Kit (Thermo Fisher, USA). Supernatant of the grinded tunica media was incubated with 50 *μ*l Amplex Red reagent (100 *μ*M)/HRP (0.2 U/ml) working solution for 30 min at 25°C, away from the light. The absorbance value of each sample was obtained at 560 nm by a spectrophotometer. In order to visualize the ROS in the aorta, the frozen sections were incubated with 20 *μ*M dihydroethidium operating fluid (Vigorous Biotechnology, China) at room temperature for 70 min. After the above procedures, the slides were mounted with Vectashield Mounting Medium with DAPI (Vector Laboratories, USA). All the fluorescence images were taken by a fluorescent microscope (Olympus, Japan) at 100 or 400 magnifications. At least five areas for each slide were randomly selected for quantitative analysis via ImageJ.

### 2.7. Cell Culture and Treatments

Primary aortic VSMCs were isolated from male WKYs and were cultured in DMEM containing 10% (*v*/*v*) FBS (Gibco, USA), 100 U/ml penicillin, and 100 *μ*g/ml streptomycin at 37°C in a humidified atmosphere containing 5% CO_2_/95% room air. VSMCs between 3rd and 6th generations were adopted for the following experiments.

When VSMCs reached 70-80% confluence, they experienced a 24 h starvation in DMEM without FBS. After that, cells in the control group were maintained in DMEM and 10% FBS for 48 h. The intervened cells were treated with Ang II in DMEM and 10% FBS for 24 h, and then cultured in DMEM and 10% FBS with Ang II along or a mixture containing Ang II and ATX for another 24 h.

### 2.8. Cell Viability Assay

Cell viability was measured by Cell Counting Kit-8 (CCK-8, Biosharp, China). According to instructions, 10 *μ*l working solution was added to each well and incubated at 37°C for 2 h. The absorption values were quantified by an ELx800 Universal Microplate Reader (BioTek) at 450 nm.

### 2.9. Cell Scratch Wound Assay

2 × 10^5^ VSMCs were seeded in 6-well plates. After 24 h starvation, VSMCs were scratched using a 200 *μ*l pipette tip and washed with PBS for 2 times. Then, the VSMCs were treated as the process above. Photos were taken every 12 h. Cell migration was quantified by the ratio of healing areas at 48 h to original injured areas.

### 2.10. Transwell Migration Assay

The migratory capability of VSMCs was detected by transwell chambers (8 *μ*m pores, Millipore, USA). In short, after the treatment above, cells were digested, resuspended, and then planted on the surface of upper chambers. DMEM with 20% FBS was injected into the lower chambers to stimulate VSMC migration. After 8 h incubation, membranes at the subjacent sides of upper chambers were fixed with 4% PFA for 30 min and then stained with 0.1% crystal violet stain solution (Solarbio, China) for 20 min. Photographs were taken by an optical microscope (Leica DM3000, Germany), and the cell numbers were manually counted.

### 2.11. Measurement of Cell Cycle

Cell cycle was measured by cell cycle and apoptosis analysis kit (Beyotime, China). VSMCs were collected and fixed in 70% ethanol at 4°C for 14 h. Then, the fixed cells were incubated with propidium iodide (PI) and RNase A staining solution in the dark at 37°C for 30 min. DNA content analyses were performed by FACSCalibur flow cytometer (BD Biosciences, USA), and the obtained cell cycle data were analyzed with FlowJo software.

### 2.12. Fluorescence Measurements in Cultured VSMCs

1 × 10^6^ VSMCs were inoculated in confocal dishes. All the following fluorescence images were captured by a confocal microscope (Olympus, Japan) and were quantitatively analyzed via ImageJ. EdU-positive VSMCs were detected by EdU Cell Proliferation Kit (Beyotime, China). Cells were incubated with EdU working solution (10 mM) for 2 h and then stained according to operation specification. TUNEL-positive VSMCs were detected by TUNEL Apoptosis Assay Kit (Beyotime, China). Cells were fixed, permeabilized, and then incubated with TUNEL reaction mixture at dark for 60 min. Calcium concentration was measured by the following procedures. VSMCs were washed by Hanks' balanced salt solution (HBSS) for 3 times and loaded with 10 *μ*M Fluo-4/AM working solution at 37°C for 20 min. Then, the VSMCs were washed with HBSS for 3 times, and 1 ml of HBSS was left to cover the cells. The image of calcium in each group was continually observed and photographed for 400 sec. At 50 sec, 50 *μ*l of KCl was added to stimulate calcium release. The initial fluorescence intensity of image was detected as F0. The fluorescence intensity at different time points was detected as FI. The relative concentration changes of calcium were calculated as ΔF/F0 = (FI − F0)/F0. Cellular ROS and mitochondrial ROS (mtROS) were determined by reactive oxygen species assay kit (Beyotime, China) and mitochondrial superoxide indicator (Molecular Probes, USA). Having been washed by HBSS for 2 times, the VSMCs were incubated with fluorescent probe indicators at dark for 20 min, and then washed for 3 times. The ROS and mtROS contents were represented as the area of intensity (AOI) of red fluorescent. Mitochondrial membrane potential (MMP) was measured by mitochondrial membrane potential assay kit (JC-1, Beyotime, China). The cells were incubated with 2 *μ*M JC-1 probe at dark for 30 min. MMP was calculated as the ratio of red to green fluorescence intensity according to the instruction.

### 2.13. Enzymatic Activities and the Content of MDA

The total proteins of VSMCs were extracted in RIPA lysis buffer (Solarbio, China) at 4°C, followed by 5 min centrifugation at 12,000 rpm to remove cell fragments. The concentration of protein was measured by BCA Protein Assay Kit (Beyotime, China). The activities of NADPH oxidase and xanthine oxidase were assayed by NADPH oxidase activity quantitative assay kit (Genmed Scientifics Inc., USA) and xanthine oxidase assay kit (Nanjing Jiancheng Bioengineering Institute, China). The activities of SOD and Mn-SOD were determined using CuZn/Mn-SOD assay kit (Beyotime, China). The content of MDA was assessed via MDA assay kit (Nanjing Jiancheng Bioengineering Institute, China). The values of these indexes were detected according to their instructions and were standardized by protein contents.

### 2.14. Bioenergetic Quantification of Mitochondrial Oxygen Consumption Rate (OCR)

**Figure 1:**
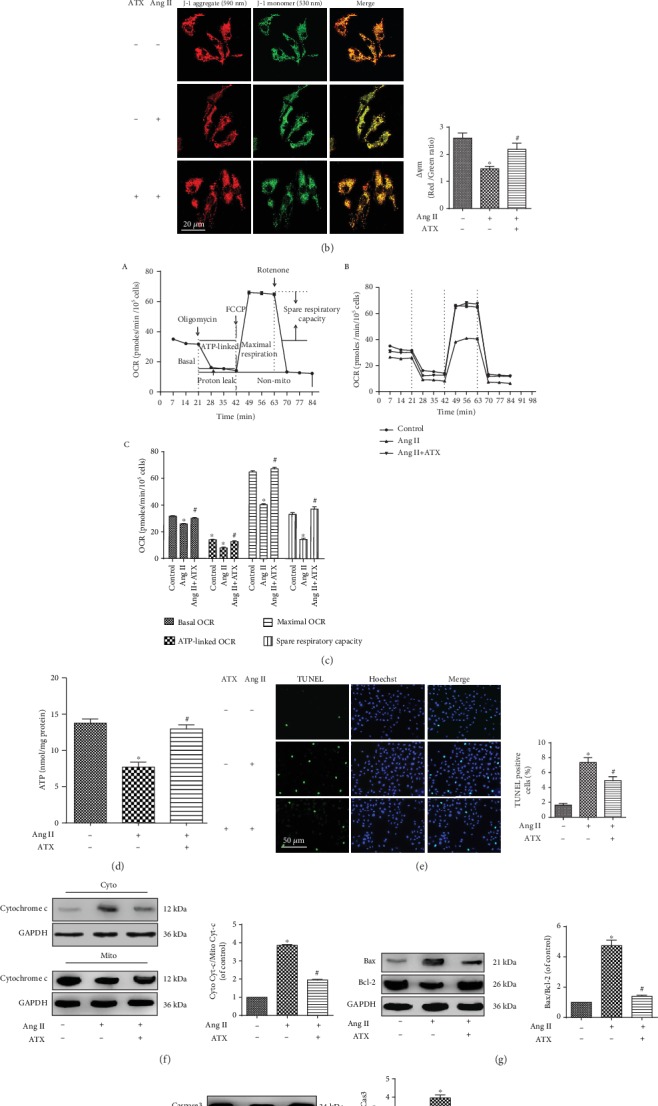
ATX improves Ang II-induced mitochondrial dysfunction in VSMCs. (a) The fluorescence intensity of Ca^2+^ concentration in VSMCs. The relative values were shown as the comparison between the peak and the baseline fluorescence intensity. The fluorescence intensities of Ca^2+^ concentration in experimental groups were calculated to draw the time-course curves. Bar = 20 *μ*m. (b) MMP of VSMCs was imaged by confocal microscopy. The orange fluorescence represents the superposition of green fluorescence (JC-1 monomer) and red fluorescence (JC-1 aggregate). The mitochondrial MMP in each group was quantified. Bar = 20 *μ*m. (c) Evaluation of VSMCs mitochondrial bioenergy metabolism function. (A) Schematic general view of mitochondrial oxygen consumption rate (OCR), including basal OCR, ATP-related OCR, maximal respiration, proton leak, spare respiratory capacity, and nonmitochondrial OCR. (B) Analyses of mitochondrial OCRs data in real-time. (C) Quantitative analyses of the impact of ATX on mitochondrial OCRs. (d) Impacts of ATX on the decline of cellular ATP generation. (e) Cell apoptosis was assessed by TUNEL staining, and the percentage of TUNEL-positive VSMCs was quantified. Bar = 50 *μ*m. The protein expressions of cytoplasm and mitochondria cytochrome C (f), Bax, Bcl-2 (g) as well as cleaved caspase 3 (h) were analyzed by Western blot analysis. Data are represented as mean ± SEM of 6 independent experiments. ^∗^*P* < 0.05 vs. untreated controls; ^#^*P* < 0.05 vs. VSMCs induced by Ang II (1 *μ*M).

The extracellular flux 96 (XF96) analyzer (Seahorse Biosciences, Agilent Technologies, USA) was applied to measure mitochondrial respiration of VSMCs. After the treatment above, the culture medium in each group was replaced by XF96 analyzer medium 1 h before the test. As shown in Figure 1(d), the basal OCR was measured at 7 min of the test. At 21 min, 2 *μ*M oligomycin was added, after which the ATP-linked OCR was detected. Then after the uncoupling effect of 2 *μ*M FCCP, the maximal OCR was captured. At last, spare respiratory capacity was calculated after the intervention of 1 *μ*M rotenone. All the values were standardized by the cell number of each well.

### 2.15. Detection of ATP Content

Intracellular ATP content was measured via luciferase-based luminescence method. Briefly, the VSMCs were disrupted in lysis buffer at 4°C and centrifuged for 5 min at 12,000 rpm. The supernatant of each group was harvested and then added to the ATP detection working solution (Beyotime, China). The values of luminescence were read by a luminometer (Turner BioSystems, USA) immediately after the processes above and were standardized by protein contents.

### 2.16. Transmission Electron Microscopy (TEM)

VSMCs were digested, centrifuged, and fixed by glutaraldehyde overnight. Subsequently, the specimens were fixed by 1% osmium tetroxide for 1 h, progressively dehydrated with rising concentrations of ethanol, and finally embedded in TAAB Epon (Marivac, Canada). The ultrathin sections (60 nm) were prepared on copper grids and stained with 1% uranyl acetate and lead citrate. Images were taken under TEM (Tecnai G2 Spirit BioTWIN; FEI, USA) at an accelerating voltage of 80 kV. Mitochondrial parameters including length, width, area, and length to width ratio were measured using MetaMorph software in five randomly selected fields. The mitophagy number was counted in the whole cell.

### 2.17. Western Blot Analysis

The total proteins of VSMCs were extracted in RIPA lysis buffer (Solarbio, China) at 4°C, followed by 5 min centrifugation at 12,000 rpm to remove cell fragments. To extract cytosolic and mitochondrial proteins, the VSMCs were rinsed with PBS for 3 times, harvested, and lysed by mitochondrial separation reagent (Beyotime, China) on ice for 15 min. Mitochondria and cytosolic fractions were separated by centrifugations at different speeds. The extracted mitochondria were further lysed by Mitochondrial Lysis Buffer (Genmed Scientifics, USA). The concentration of protein was measured by BCA Protein Assay Kit (Beyotime, China). Proteins (10-30 *μ*g) in each group were resolved by 10% SDS-polyacrylamide gel and then transferred onto polyvinylidene difluoride membranes. After being blocked for 2 h, the membranes were incubated with the primary antibodies including anti-Bax antibody, anti-Bcl-2 antibody, anti-cytochrome C antibody, anti-cleaved caspase 3 antibody, anti-PGC-1*α* antibody, anti-Drp1 antibody, anti-Drp1 (phospho S616) antibody, anti-PINK1 antibody, anti-parkin antibody, anti-VDAC1/Porin antibody, anti-GAPDH antibody (Abcam, UK), and antibodies against Akt, p-Akt, JNK, p-JNK (Upstate Biotechnology, USA) as well as ERK1/2, p-ERK1/2, p38, and p-p38 (Cell Signaling Technology, USA) overnight at 4°C. After being rinsed off with TBS for 3 times, the membranes were incubated with anti-rabbit or anti-mouse IgG secondary antibodies that conjugated with HRP (Abcam, UK). After 1 h incubation at room temperature, the blots were detected by Pierce™ enhanced chemiluminescence (ECL) Western Blotting Substrate (Thermo Fisher Scientific, USA). The protein band intensities were calculated by Gel-Pro Analyzer software.

### 2.18. Real-Time Quantitative PCR (RT-qPCR)

The total RNAs from each sample were isolated by TRIzol reagent (Invitrogen, USA) and transcribed to complementary DNA (cDNA) by PrimeScript™ RT reagent kit (Takara, Japan). The following amplifications of cDNA were performed by SYBR Premix Ex Taq™ (Takara, Japan) under the procedure below: the first cycle was set at 95°C for 30 sec, the following 40 cycles were set for 5 sec at 95°C, then annealing at 60°C for 31 sec. The primer sequences used in mitochondrial transcription factor A (Tfam) and GAPDH were designed as follows. Primers for Tfam are 5′-ATCTCATCCGTCGCAGTG-3′ (forward primer) and 5′-GCACAGTCTTGATTCCAGTTC-3′ (reverse primer). Primers for GAPDH are 5′-GTGAAGGTCGGTGTGAAC-3′ (forward primer) and 5′-GGTGAAGACGCCAGTAGA-3′ (reverse primer). The threshold cycle (Ct) value was demanded at the set time of PCR logarithmic phase. The relative content of mRNA was calculated by the comparisons of target PCR cycle times (Ct). The Ct value of each target PCR was standardized by subtracting the Ct value of GAPDH. And the relative value of mRNA expression for each sample was obtained by following the calculation of 2-(*Δ*Ct sample-*Δ*Ct control) method. The experimental protocol above was applied to the quantitative analysis of mtDNA equally. The amplification level of mtDNA (Rnr2) was normalized against GAPDH. Primers for Rnr2 are 5′-AGCTATTAATGGTTCGTTTGT-3′ (forward primer) and 5′-AGGAGGCTCCATTTCTCTTGT-3′ (reverse primer).

### 2.19. Statistical Analysis

Data were expressed as mean ± SEM. Statistical analyses were assessed with Student's *t*-test or one-way analysis of variance (ANOVA) followed by post hoc tests. Blood pressures and vasoconstrictions were analyzed by mixed effect model for repeated measures using SPSS (version 19, IBM, USA). The EC50 and maximum response to phenylephrine and Ca^2+^ were calculated by nonlinear regression analysis using Prism software (version 6.0, GraphPad Software Inc., USA). A value of *P* < 0.05 was considered statistically significant.

## 3. Results

### 3.1. Beneficial Effects of ATX on Blood Pressure and Vascular Remodeling in SHRs

**Figure 2:**
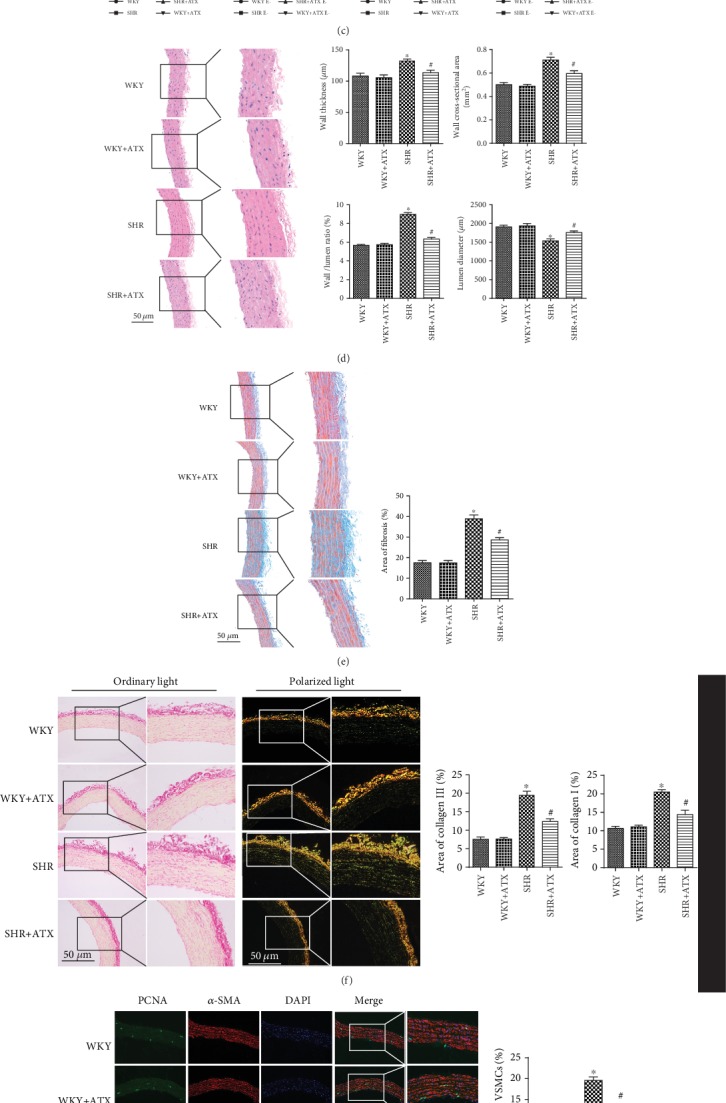
Astaxanthin (ATX) reduces blood pressure and improves aortic systolic function as well as vascular remodeling in SHRs. (a) Systolic blood pressure (SBP) and diastolic blood pressure (DBP) in each group. (b) The levels of renin, Ang II, and ALD in the serum of each group. (c) Phenylephrine-induced and Ca^2+^-induced vasoconstrictions of aortic rings with or without endothelium in each group. (d) Representative images of aortic sections in each group stained by H&E staining and quantitative analyses of the wall thickness (WT), wall cross-sectional area (WCSA), wall/lumen ratio, and lumen diameter (LD) of each group. Bar = 50 *μ*m. (e) Representative images of aortic sections in each group stained by Masson's trichrome staining and quantitative analysis of the area ratio of fibrosis in each group. Bar = 50 *μ*m. (f) Representative images of aorta sections in each group stained by Sirius-red staining and quantitative analyses of the area ratio of collagen I and collagen III in each group. Bar = 50 *μ*m. (g) Representative images of aortic sections in each group stained for PCNA (green), *α*-SMA (red), and DAPI (blue) and quantitative analysis of the percentage of PCNA-positive VSMCs. Bar = 100 *μ*m. (h) Representative images of aortic sections in each group stained for Ki67 (green), *α*-SMA (red), and DAPI (blue) and quantitative analysis of the percentage of Ki67-positive VSMCs. Bar = 100 *μ*m. Values are represented as mean ± SEM from 8 rats in each group. ^∗^*P* < 0.05 vs. corresponding WKYs; ^#^*P* < 0.05 vs. corresponding SHRs.

This study demonstrated the therapeutic effects of ATX on blood pressure in SHRs. At the beginning of this study, the average SBP and DBP in SHRs were 120 and 83 mmHg, which were obviously higher than that of WKYs. SHRs suffered from gradually elevated blood pressure rising to 185/124 mmHg at 11 weeks of the study. ATX showed persistent and stable antihypertensive effects. Compared with untreated SHRs, the SBP and DBP of ATX-treated SHRs were significantly reduced from 4 weeks of treatment to the end of this study, and their average blood pressure was only 153/100 mmHg at 11 weeks (Figure 2(a)). ATX also inhibited the overactivation of the renin-angiotensin-aldosterone system (RAAS) in serum. The serum content of renin, Ang II, and ALD was elevated in SHRs, whereas ATX significantly reduced the levels of renin, Ang II, and ALD (Figure 2(b)). To determine the effects of ATX on arterial systolic function, we detected the reactivity of aortic rings to phenylephrine and Ca^2+^. When compared with WKYs, the vascular contractility to phenylephrine and Ca^2+^ was remarkably enhanced in SHRs, with or without endothelium, in a concentration-dependent manner. On the contrary, the reactivity of aortic rings to phenylephrine and Ca^2+^ was significantly restrained by ATX (Figure 2(c), Tables S1 and S2). ATX did not affect the blood pressure, RASS activation, or vasoconstriction in WKYs.

Vascular parameters of the aorta were measured by H&E staining. Compared with WKYs, SHRs suffered from increased wall thickness (WT), wall cross-sectional area (WCSA), and wall/lumen ratio, along with decreased lumen diameter (LD). By contrast, ATX prevented vascular remodeling by reducing WT, WCSA, and wall/lumen ratio and maintaining LD in SHRs (Figure 2(d)). Attempts were then made to detect the severity of fibrosis in aortic tunica media by Masson's trichrome staining. The results showed that much more fibers deposited in SHRs than in WKYs. In comparison, the fibrosis was attenuated after the treatment of ATX (Figure 2(e)). The results were further confirmed by Sirius-red staining, in which collagen I was marked as yellow fluorescence and collagen III was labeled as green fluorescence under polarization microscope. We found the area ratios of collagen I and collagen III were much higher in SHRs when compared with WKYs, whereas ATX suppressed the deposition of collagen I and collagen III in SHRs (Figure 2(f)). VSMC proliferation was detected by double-immunofluorescence staining. The study showed highly increased ratio of PCNA-positive VSMCs and Ki67-positive VSMCs in the aorta of SHRs. After 11 weeks of ATX treatment, the ratio of PCNA-positive VSMCs and Ki67-positive VSMCs was significantly decreased (Figures 2(g) and 2(h)). To sum up, the study demonstrated ATX could lower blood pressure, reduce vascular contractility, and alleviate vascular remodeling in SHRs partly through inhibiting the proliferation of VSMCs.

### 3.2. Effects of ATX on Oxidative Stress in Serum, Tunica Media, and Aortic VSMCs of SHRs

**Figure 3:**
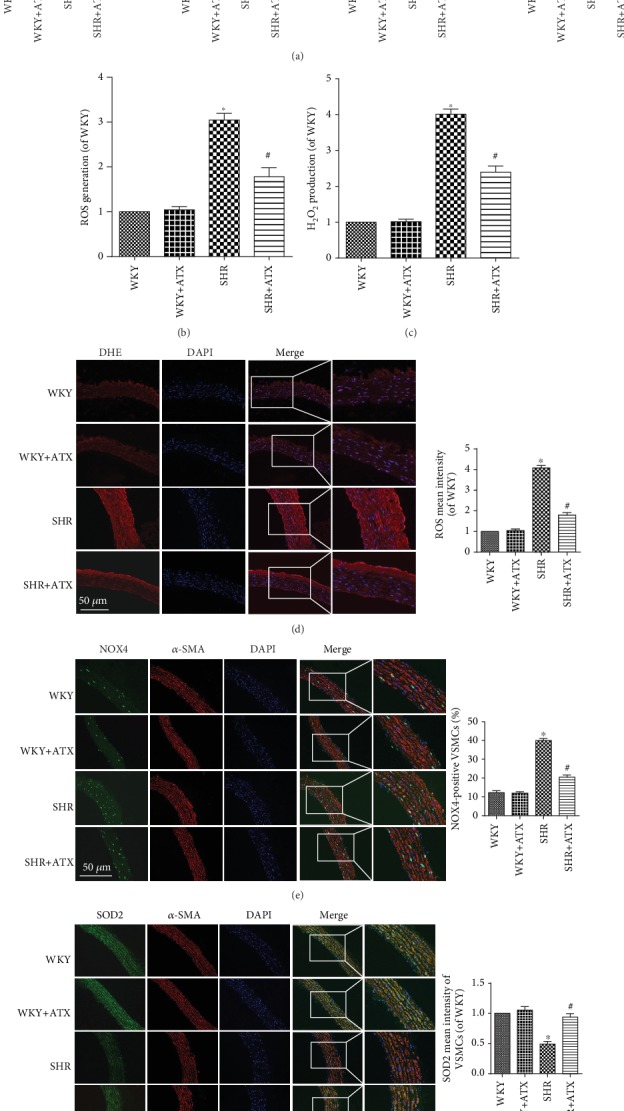
ATX reduces oxidative stress in serum, tunica media, and aortic VSMCs of SHRs. (a) The content of MDA and GSH and the enzyme activities of SOD and GSH-PX in the serum of each group. (b) The ROS levels of each group measured by lucigenin-enhanced chemiluminescence. (c) The H_2_O_2_ production of each group measured by Amplex Red. (d) Representative pictures of the ROS (red) by dihydroethidium staining and quantitative analysis of ROS mean intensity. Bar = 100 *μ*m. (e) Representative images of aortic sections in each group stained by NOX4 (green), *α*-SMA (red), and DAPI (blue) and quantitative analysis of the percentage of NOX4-positive VSMCs. Bar = 100 *μ*m. (f) Representative images of aortic sections in each group stained for SOD2 (green), *α*-SMA (red), and DAPI (blue), as well as the quantitative analysis of SOD2 mean intensity of VSMCs. Bar = 100 *μ*m. Values are represented as mean ± SEM from 8 rats in each group. ^∗^*P* < 0.05 vs. corresponding WKYs; ^#^*P* < 0.05 vs. corresponding SHRs.

Overactivation of oxidative stress plays a vital role in the emergence and development of hypertension. MDA is a lipid peroxidation product that reflexes the level of oxidative stress. GSH, SOD, and GSH-PX are the three major antioxidants. SHRs showed higher level of oxidative stress in serum with distinctly increased MDA content, reduced GSH content as well as decreased enzyme activities of SOD and GSH-PX. However, the adverse outcomes in SHRs were remarkably reversed by the addition of ATX (Figure 3(a)). Later, oxidative stress in tunica media and VSMCs was detected. Fluorescence staining demonstrated the ROS mean intensity in tunica media, and the ratio of NOX4-positive VSMCs was increased in SHRs, which were accompanied by reduced SOD2 mean intensity (Figures 3(d)–3(f)). In addition, the ROS level and the H_2_O_2_ content in tunica media were also increased (Figures 3(b) and 3(c)). After 11 weeks treatment of ATX, the ROS mean intensity, ROS level, H_2_O_2_ content, and NOX4-positive VSMCs were notably declined, and the SOD2 mean intensity was significantly increased in SHRs (Figures 3(b)–3(f)). The indexes of oxidative stress in WKYs were not changed after ATX intervention. Taken together, the above results suggested that ATX can inhibit the overactivation of oxidative stress and stabilize the balance between prooxidants and antioxidants.

### 3.3. The Antiproliferative Effects of ATX on Cultured VSMCs

**Figure 4:**
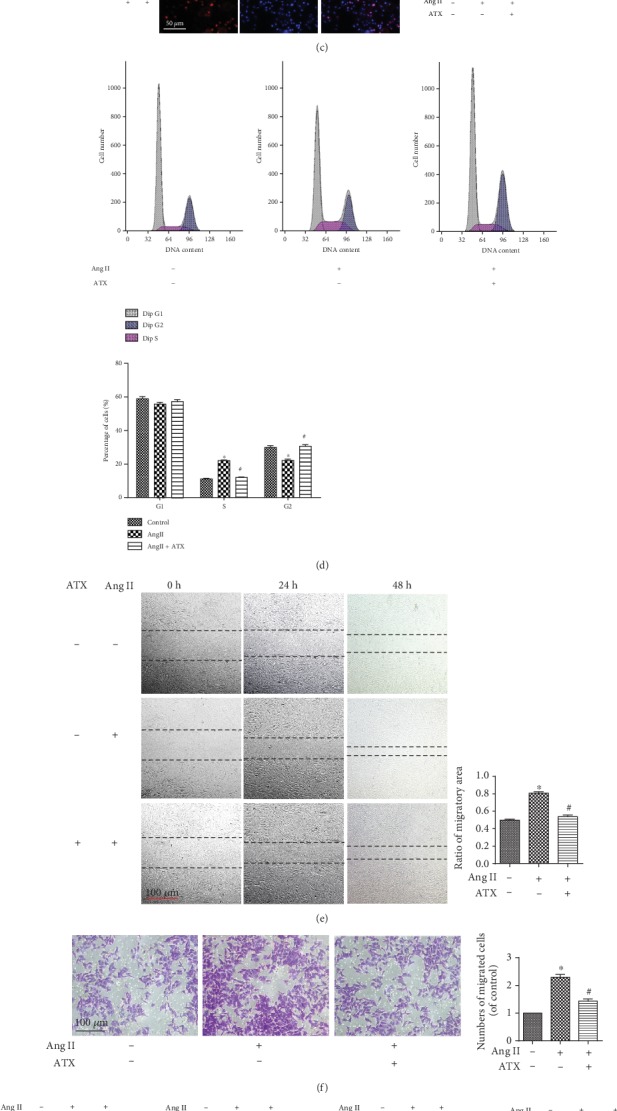
ATX inhibits the proliferation of VSMCs. (a) Cells were treated with progressive increased concentrations of Ang II (0-10 *μ*M) for 24 h, cell viability in each group was assessed by CCK-8 kit. (b) The VSMCs were disposed to Ang II for 24 h and then treated with Ang II along or a mixture containing Ang II and increasing concentration of ATX (10, 15, 20, and 25 *μ*M) for another 24 h. Cell viability in each group was assessed by CCK-8 kit. (c) Cell proliferation was assessed by EdU staining, and the percentage of EdU-positive VSMCs was quantified. Bar = 50 *μ*m. (d) Representative samples of cell cycle progression in each experimental group and the quantitative comparison in each phase of VSMCs. (e) The wound healing was observed every 12 h and quantitative analysis of the ratio of migratory area in each group at 48 h. Bar = 100 *μ*m. (f) Transwell assays in each experimental group and the numbers of migrated cells were quantified. Bar = 100 *μ*m. (g) The protein expressions of p38, ERK1/2, JNK, and Akt were analyzed by Western blot analysis. Values are represented as mean ± SEM of 6 independent experiments. ^∗^*P* < 0.05 vs. untreated controls; ^#^*P* < 0.05 vs. VSMCs injured by Ang II (1 *μ*M).

In this work, we used Ang II to stimulate the proliferation of cultured VSMCs. CCK-8 assay showed that the stimulative effects of Ang II presented a positive dose-response relationship with the proliferation of VSMCs and reached plateau when the concentration was 1 *μ*M. The same method was adopted to detect the effect of ATX and found ATX could reduce Ang II-related proliferation in a concentration-dependent manner, reaching a maximal inhibition at 20 *μ*M (Figures 4(a) and 4(b)). To revalidate the proliferative effect of 1 *μ*M Ang II and the antiproliferative effect of 20 *μ*M ATX, cell proliferation was assessed again by EdU staining kit and cell cycle assay. Compared with the control group, the ratio of EdU-positive cells was significantly increased in response to 1 *μ*M Ang II, accompanied by a higher percentage of cells activated in the S phase of cell cycle. In contrast, the above effects were obviously attenuated by 20 *μ*M ATX (Figures 4(c) and 4(d)). Then, cell migration was assessed by cell scratch wound assay and transwell migration assay. As expected, the migration in the Ang II group was significantly enhanced with quicker healing velocity and more migrated cells. On the contrary, the healing speed was slower and the migrated cells were less in the ATX group (Figures 4(e) and 4(f)). After that, efforts were made to evaluate the effects of ATX on MAPK and Akt pathways. Western blotting analyses demonstrated that the phosphorylation levels of p38, ERK1/2, JNK, and Akt were significantly higher in Ang II-injured cells, whereas ATX degraded the phosphorylation of these four proteins (Figure 4(g)). Overall, these data suggested that 1 *μ*M Ang II can promote VSMC proliferation and migration, while 20 *μ*M ATX displays suppressive effects on Ang II-induced pathologic changes through directly deactivating MAPK and Akt signal pathways.

### 3.4. Protective Effects of ATX on Oxidative Stress Induced by Ang II in Cultured VSMCs

**Figure 5:**
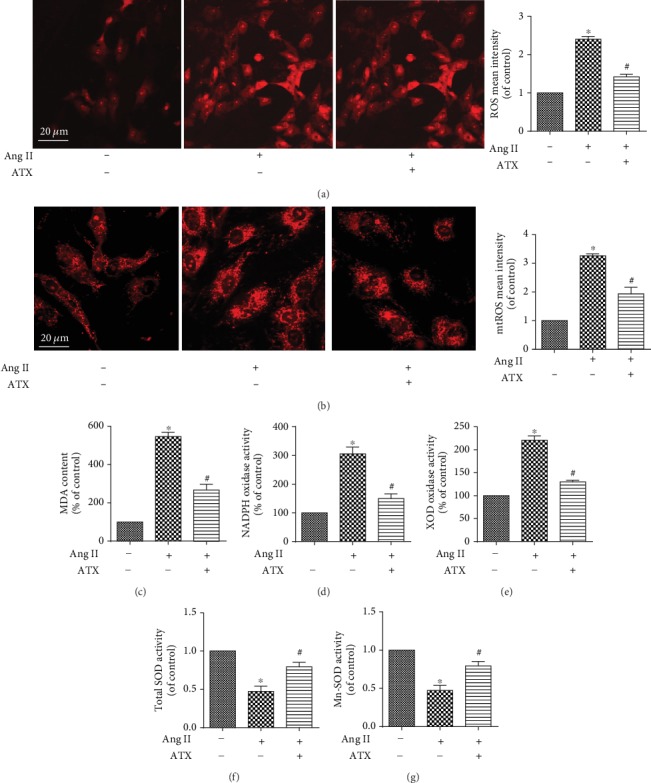
ATX decreases oxidative stress induced by Ang II in VSMCs. (a, b) Cellular ROS and mtROS of VSMCs were imaged by confocal microscopy, and the mean intensity of each group was quantified. Bar = 20 *μ*m. The content of MDA (c) and the enzymatic activities of NADPH oxidase (d), xanthine oxidase (e), SOD (f), and Mn-SOD (g) of VSMCs in each group were analyzed. Values are represented as mean ± SEM of 6 independent experiments. ^∗^*P* < 0.05 vs. untreated controls; ^#^*P* < 0.05 vs. VSMCs injured by Ang II (1 *μ*M).

Attempts were then made to investigate the oxidative stress injuries in cultured VSMCs. The Ang II-injured VSMCs exhibited a significant enhancement in the aggregation of cellar ROS, mtROS, and MDA (Figures 5(a)–5(c)), indicating higher oxidative stress level. Additionally, VSMCs subjected to Ang II showed higher enzymatic activities of NADPH oxidase and xanthine oxidase, along with overtly subdued SOD activity (Figures 5(d)–5(f)). What is more, Mn-SOD that plays a crucial role in regulating mtROS generation also suffered from downregulation (Figure 5(g)). The treatment of ATX evidently attenuated the above effects of Ang II, decreases of ROS, mtROS, and MDA, as well as reduced enzymatic activities of NADPH oxidase and xanthine oxidase were found in ATX-treated cells, along with improved SOD and Mn-SOD activities (Figures 5(a)–5(g)). Taken together, these results confirmed that ATX possesses the abilities to antagonize Ang II-induced oxidative stress injuries by lowering ROS accumulation and regulating related enzymatic activities.

### 3.5. Protective Effects of ATX on Ang II-Induced Mitochondrial Dysfunction in Cultured VSMCs

Abnormal accumulation of ROS leads to lipid peroxidation of cell membrane, resulting in cellular Ca^2+^ overload and mitochondrial dysfunction. In the present study, Ang II induced severe Ca^2+^ overload in VSMCs. After adding 50 *μ*l KCl to stimulate calcium release, the relative fluorescence intensity of calcium reached peak at 120 sec, and the maximum *Δ*F/F0 was 2.1 fold of the undamaged cells (Figure 1(a)). What is more, the mitochondrial membrane potential (MMP) and cellular ATP content were significantly reduced in VSMCs exposed to Ang II (Figures 1(b) and 1(d)). Bioenergetic quantification of mitochondrial oxygen consumption rate (OCR) was performed later to clarify the influence of Ang II on mitochondrial energy transfer. According to our findings, the mitochondrial OCRs were significantly decreased in Ang II-injured VSMCs as compared with the control group (0.82-fold decrease in basal OCR, 0.56-fold decrease in ATP-linked OCR, 0.62-fold decrease in maximal OCR, and 0.44-fold decrease in spare respiratory capacity, separately). On the contrary, ATX remarkably inhibited cellular Ca^2+^ overload. The maximum *Δ*F/F0 was reduced by half when compared with Ang II-injured cells (Figure 1(a)). Furthermore, ATX-treated VSMCs showed improved mitochondrial functions with increased MMP, cellular ATP content, and mitochondrial OCRs (Figures 1(b)–1(d)). The present study also found apoptosis was increased in Ang II-injured cells, along with increased cytoplasmic cytochrome C (Cyt-c) to mitochondrial Cyt-c ratio, Bax to Bcl-2 ratio, and cleaved-caspase 3 to caspase 3 ratio. In contrast, the apoptosis was significantly decreased after ATX treatment. Furthermore, the release of cytochrome C, the modulation of Bax/Bcl2 protein, and the activation of caspase 3 were inhibited by ATX treatment (Figures 1(e)–1(h)).

### 3.6. Protective Effects of ATX on Ang II-Induced Mitochondrial Structural Damages, Mitophagy, and Mitochondrial Biosynthesis

**Figure 6:**
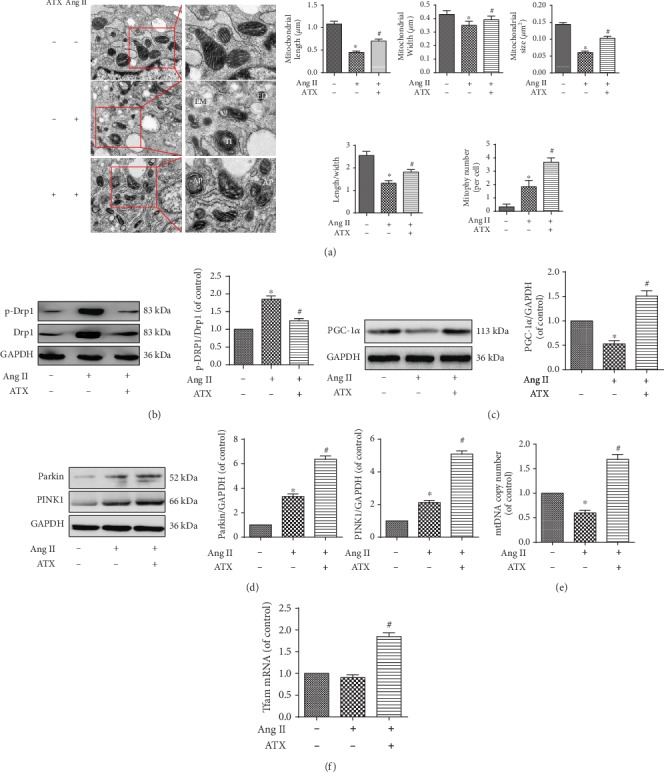
ATX alleviates Ang II-induced mitochondrial structural damages of VSMCs and regulates the expression of genes and proteins that associated with mitochondrial fission, mitophagy, and mitochondrial biosynthesis. (a) Mitochondrial ultrastructure features of VSMCs under transmission electron micrographs (TEM) and the length, width, area, and length to width ratio of mitochondria, as well as mitophagy number in each group. VD: vacuolar deformation; TI: thread-like intima; ED: electron-densed depositions; AP: autophagosome. Bar = 500 nm. Protein expressions of Drp1 and p-Drp1 (b), PGC-1*α* (c), and parkin and PINK1 (d). Expression of mtDNA (e) copy member and Tfam mRNA (f). Data are represented as mean ± SEM of 6 independent experiments. ^∗^*P* < 0.05 vs. untreated controls; ^#^*P* < 0.05 vs. VSMCs induced by Ang II (1 *μ*M).

Morphological changes of mitochondria in cultured VSMCs were detected by TEM. Mitochondria in the control group were intact, the cristae and matrices were clear, distinguishable, and in order (Figure 6(a)). After being damaged by Ang II for 48 h, the mitochondrial cristae were swollen, deformed, or decreased, and some mitochondria even suffered from vacuolar deformation, electronic intensity deposits in matrices, and spiral defects in mitochondrial intima. The mitochondrial length, width, area, and the length to width ratio were reduced, suggesting enhanced mitochondrial fission (Figure 6(a)). These pathological changes were significantly attenuated in the ATX group; the small and disorganized mitochondria were less to be found (Figure 6(a)). Mitophagy is a special form of autophagy and shown as a number of damaged mitochondria that were encircled by multilayer structures in Figure 6(a). In the present study, more mitophagy was found in the ATX group when compared with the Ang II group (Figure 6(a)).

The results of TEM showed that ATX inhibited mitochondrial fission and stimulated mitophagy in Ang II-injured VSMCs. Therefore, further attempts were then made to detect the related proteins by Western blotting. Drp1 is a critical regulator of fission; the protein content of Drp-1 and the ratio of p-Drp1 (Ser616) to Drp1 were significantly increased after Ang II. Conversely, ATX showed deterrent effects on its protein expression and phosphorylation (Figure 6(b)). Parkin and PINK1 are two mitophagy-related proteins. In our work, Ang II showed promotive effects on these two proteins, whereas ATX exerted much stronger driving force on the expression of them (Figure 6(d)). Next, we wonder whether ATX could act on mitochondrial biogenesis. The results revealed that mtDNA copy number and the protein expression of PGC-1*α* were significantly reduced in Ang II-injured cells, whereas Tfam was not affected in RNA level (Figures 6(c), 6(e), and 6(f)). In contrast, ATX increased the mtDNA copy number, the protein expression of PGC-1*α*, and the RNA expression of Tfam (Figures 6(c), 6(e), and 6(f)). The above results indicated that ATX could repair mitochondrial structural damages, inhibit mitochondrial fission, and stimulate mitophagy and mitochondrial biogenesis.

## 4. Discussion

In the present study, we investigated the protective effects of ATX against hypertension in SHRs and Ang II-injured VSMCs. Our data suggested that ATX may attenuate hypertension in terms of reduced blood pressure and alleviated aortic remodeling partly through inhibiting VSMCs from proliferation via mitigating oxidant stress and improving mitochondrial functions. What is more, ATX could regulate mitochondrial quality control measures via inhibiting mitochondrial fission and stimulating mitophagy and mitochondrial biogenesis. These results may be essential for a better understanding of the molecular mechanisms of ATX.

In hypertension, VSMCs in the conduit arteries undergo transition from a contractile phenotype to a more synthetic phenotype in response to vascular wall stress and vasoactive substances. The activation of the synthetic phenotype not only makes VSMCs themselves proliferating and hypertrophying but also accelerates extracellular matrix deposition, resulting in vascular dysfunction and remodeling [3]. Previous studies found that ATX could lower the blood pressure in SHRs without affecting the blood pressure of normal rats [14–16]. Nevertheless, the protective effects and mechanisms within the pathological improvement on the aorta were not expounded in details. In the present study, we found that ATX lowered the ratio of PCNA-positive VSMCs and ki67-positive VSMCs in the aorta, prevented cells from reentering cell cycle, and restrained the migratory abilities in Ang II-injured VSMCs. These data supported that VSMCs were targets for ATX to alleviate vascular remodeling.

In hypertension, superoxide free radicals and NADPH oxidase could serve as stimulating factors for the proliferation of VSMCs and contribute to the development of arterial remodeling. As a ROS scavenger and chemically antioxidant, ATX has two special structures, the polyene chain and terminal ring, which equipped ATX with outstanding antioxidant capacity to capture and scavenge radicals and prevent chain reactions. Meanwhile, ATX has been suggested to improve SOD and glutathione and inhibit the xanthine oxidase and NADPH oxidase, therefore balance the prooxidant and antioxidant [20]. In the present study, oxidant load and poor defensive ability to oxidative stress of VSMCs in the aorta were alleviated by ATX with reduced ROS, more expression of SOD2, and less expression of NOX4 and H_2_O_2_. These results were verified in Ang II-injured VSMCs. ATX reduced ROS and restored the activities of GSH-PX, NADPH oxidase, xanthine oxidase, SOD, and Mn-SOD. The above results suggested that ATX might attenuate the progression of vascular remodeling via inhibiting the proliferation of VSMCs and alleviating oxidative stress injuries.

In oxidative stress-associated diseases, mitochondria are more likely to be dysfunctional because they are both the source and the victim of toxic-free radicals [21]. Generally, in the process of oxidative stress, altered calcium homeostasis is regarded as the initial factor for mitochondrial injuries [22]. That was followed by mitochondrial polarization with the disruption of MMP. Mitochondrial polarization represents mitochondrial dysfunction and affects the ATP generation by weakening the mitochondrial electron transport chain and metabolic oxygen consumption [23]. In the present studies, ATX inhibited the accumulation of cellular ROS and mtROS and kept the calcium homeostasis in Ang II-injured VSMCs. What is more, the MMP was improved, the mitochondrial OCRs were recovered, and the ATP content was increased after ATX treatment. Mitochondria-associated apoptosis was another important pattern of mitochondrial dysfunction. In a previous study, VSMCs suffered from ROS-dependent apoptosis after Ang II injury, and this is mainly caused by the release of Cyt-c and the activation of mitochondria apoptosis pathway [24]. Astaxanthin was shown to inhibit cytochrome C release resulting from mitochondria permeabilization and thereby prevent mitochondria-mediated apoptotic death in cardiomyocytes and endotheliocyte [25]. Accordingly, our work demonstrated that ATX could inhibit mitochondria-associated apoptosis via suppressing the release of Cyt-c, the protein ratio of Bax to Bcl2, and the activation of caspase 3.

Mitochondria can mitigate structural and functional damages through quality control measures by three main ways: mitochondrial dynamics, mitophagy, and mitochondrial biogenesis [8]. Mitochondrial dynamics include fission and fusion; the mitochondrial morphology changes between elongated interconnected networks and a fragmented disconnected arrangement. Abnormally enhanced mitochondrial fission was found in proliferative VSMCs [10]. One of the key guanosine triphosphatases regulating mitochondrial fission is Drp1. During fission, Drp1 is recruited from the cytoplasm to combine its mitochondrial membrane receptor Fis1. Then, Drp1 constricts and divides the mitochondria [26, 27]. Abnormally expressed Drp1 has been observed in various cardiac damages including ischemia-reperfusion injury and dilated cardiomyopathy [28, 29]. Besides, accumulated evidences suggested that Drp1-dependent mitochondrial fragmentation is closely associated with its phosphorylation status at different amino acid sites, and the phosphorylation of Ser616 was highly related to mitochondrial fission [29]. In the present study, ATX inhibited mitochondrial fragmentation and reversed the abnormal expression and phosphorylation of Drp1 in Ang II-injured VSMCs. What is more, ATX stimulated mitophagy to obliterate the dysfunctional mitochondria. Mitophagy is principally regulated by PINK1 and Parkin which work together to ubiquitinate mitochondria and activate LC3 pathway to stimulate mitophagy [30]. Mice that are deficient in Parkin or Pink1 suffer from impaired mitophagy and disorganized small mitochondria, which result in cardiac injuries and exacerbated cardiovascular diseases [31, 32]. We found mitophagy as well as the expression of PINK1 and Parkin was increased in Ang II-injured cells, which was in accordance with previous studies and could be explained by the possible fact that decreased MMP of the daughter mitochondria from enhanced fission can stabilize PINK1 on the outer membrane of depolarized mitochondria and simulate PINK1/Parkin-mediated mitophagy [33]. By contrast, ATX was more powerful to activate PINK1 and Parkin pathway and stimulate mitophagy. Mitochondrial biogenesis is another way to maintain mitochondrial homeostasis. PGC-1*α* and its downstream coactivator Tfam are two positive regulators for mitochondrial biogenesis and perform several functions for mtDNA, including enhancing mtDNA replication, repairing mtDNA, and keeping the integrity of mtDNA [34]. Moreover, studies also found PGC-1*α* can control oxidative stress response [35]. In our present study, we found treatment with ATX could significantly increase mtDNA copy number and enhance the expression of Tfam and PGC-1*α*, which further confirmed that ATX could maintain mitochondrial function by stimulating mitochondrial biogenesis. The above results provided a connection between the protective effects of ATX and the activation of mitochondrial quality control measures in hypertension and oxidative stress-induced VSMC injuries.

Despite more and more evidence demonstrating the superiority of ATX in treating cardiovascular diseases, the mechanisms are still not fully understood [36]. In recent years, very few studies were carried out to explore the protective effects of ATX on mitochondria in cardiovascular field [12]. Pongkan et al. found ATX can alleviate mitochondria swelling and mitochondria depolarization in mice hearts that suffered from ischemia-reperfusion injury [37]. Fan et al. demonstrated that ATX reduced mtROS and mitochondrial fragmentation in H9c2 cells exposed to homocysteine [25]. The current work enriched the comprehensive mechanisms of ATX and proved that ATX might exert mitochondrial benefits on hypertension by regulating mitochondrial quality control measures. To our best knowledge, this is the first work explored the favorable role of ATX in hypertension through targeting mitochondrial protection in VSMCs and implied that ATX might be beneficial in conditions characterized by oxidative stress-induced mitochondrial dysfunction. Additionally, whether ATX has the same effects or more different mechanisms on resistance vessels including mesenteric and renal arteries is another interesting subject. Further studies are needed to testify our assumption.

In conclusion, this study clearly demonstrated that treatment with ATX attenuated high blood pressure and alleviated vascular remodeling in SHRs. The protective effects of ATX may at least partially depend on the protection of mitochondrial function by regulating mitochondrial dynamics, mitophagy, and biogenesis. These findings suggested an attractive novel strategy of ATX for the treatment of a number of pathological states featured with oxidative stress-induced mitochondrial dysfunction.

## Data Availability

The data that support the findings of this study are available from the corresponding author upon reasonable request.

## Supplementary Materials

Supplementary Materials Table S1: EC50 analyses for phenylephrine and Ca^2+^-induced vasocontractions. Table S2: maximum response analyses for phenylephrine and Ca^2+^-induced vasocontractions.

## References

[1] Touyz R. M., Alves-Lopes R., Rios F. J. (2018). Vascular smooth muscle contraction in hypertension. *Cardiovascular Research*.

[2] Owens G. K. (1995). Regulation of differentiation of vascular smooth muscle cells. *Physiological Reviews*.

[3] Shi N., Chen S. Y. (2016). Smooth muscle cell differentiation: model systems, regulatory mechanisms, and vascular diseases. *Journal of Cellular Physiology*.

[4] Lacolley P., Safar M. E., Regnault V., Frohlich E. D. (2009). Angiotensin II, mechanotransduction, and pulsatile arterial hemodynamics in hypertension. *American Journal of Physiology. Heart and Circulatory Physiology*.

[5] Su B., Mitra S., Gregg H. (2001). Redox regulation of vascular smooth muscle cell differentiation. *Circulation Research*.

[6] Clempus R. E., Sorescu D., Dikalova A. E. (2007). Nox4 is required for maintenance of the differentiated vascular smooth muscle cell phenotype. *Arteriosclerosis, Thrombosis, and Vascular Biology*.

[7] Martin-Garrido A., Brown D. I., Lyle A. N. (2011). NADPH oxidase 4 mediates TGF-*β*-induced smooth muscle *α*-actin via p38MAPK and serum response factor. *Free Radical Biology and Medicine*.

[8] Baker M. J., Tatsuta T., Langer T. (2011). Quality control of mitochondrial proteostasis. *Cold Spring Harbor Perspectives in Biology*.

[9] Wang L., Yu T., Lee H., O'Brien D. K., Sesaki H., Yoon Y. (2015). Decreasing mitochondrial fission diminishes vascular smooth muscle cell migration and ameliorates intimal hyperplasia. *Cardiovascular Research*.

[10] Chalmers S., Saunter C., Wilson C., Coats P., Girkin J. M., McCarron J. G. (2012). Mitochondrial motility and vascular smooth muscle proliferation. *Arteriosclerosis, Thrombosis, and Vascular Biology*.

[11] Andreux P. A., Houtkooper R. H., Auwerx J. (2013). Pharmacological approaches to restore mitochondrial function. *Nature Reviews Drug Discovery*.

[12] Kim S., Kim H. (2018). Inhibitory effect of astaxanthin on oxidative stress-induced mitochondrial dysfunction-a mini-review. *Nutrients*.

[13] Fakhri S., Abbaszadeh F., Dargahi L., Jorjani M. (2018). Astaxanthin: a mechanistic review on its biological activities and health benefits. *Pharmacological Research*.

[14] Hussein G., Goto H., Oda S. (2005). Antihypertensive potential and mechanism of action of astaxanthin: II. Vascular reactivity and hemorheology in spontaneously hypertensive rats. *Biological & Pharmaceutical Bulletin*.

[15] Hussein G., Goto H., Oda S., Sankawa U., Matsumoto K., Watanabe H. (2006). Antihypertensive potential and mechanism of action of astaxanthin: III. Antioxidant and histopathological effects in spontaneously hypertensive rats. *Biological & Pharmaceutical Bulletin*.

[16] Monroy-Ruiz J., Sevilla M. A., Carron R., Montero M. J. (2011). Astaxanthin-enriched-diet reduces blood pressure and improves cardiovascular parameters in spontaneously hypertensive rats. *Pharmacological Research*.

[17] Islam M. A., al Mamun M. A., Faruk M. (2017). Astaxanthin ameliorates hepatic damage and oxidative stress in carbon tetrachloride-administered rats. *Pharmacognosy Research*.

[18] Ni Y., Nagashimada M., Zhuge F. (2015). Astaxanthin prevents and reverses diet-induced insulin resistance and steatohepatitis in mice: a comparison with vitamin E. *Scientific Reports*.

[19] Park J. S., Mathison B. D., Hayek M. G., Zhang J., Reinhart G. A., Chew B. P. (2013). Astaxanthin modulates age-associated mitochondrial dysfunction in healthy dogs. *Journal of Animal Science*.

[20] Fang Q., Guo S., Zhou H., Han R., Wu P., Han C. (2017). Astaxanthin protects against early burn-wound progression in rats by attenuating oxidative stress-induced inflammation and mitochondria-related apoptosis. *Scientific Reports*.

[21] Stepien K. M., Heaton R., Rankin S. (2017). Evidence of oxidative stress and secondary mitochondrial dysfunction in metabolic and non-metabolic disorders. *Journal of Clinical Medicine*.

[22] Toledo F. D., Perez L. M., Basiglio C. L., Ochoa J. E., Sanchez Pozzi E. J., Roma M. G. (2014). The Ca^2+^-calmodulin-Ca^2+^/calmodulin-dependent protein kinase II signaling pathway is involved in oxidative stress-induced mitochondrial permeability transition and apoptosis in isolated rat hepatocytes. *Archives of Toxicology*.

[23] Nickel A., Kohlhaas M., Maack C. (2014). Mitochondrial reactive oxygen species production and elimination. *Journal of Molecular and Cellular Cardiology*.

[24] Luo H. M., Wu X., Liu W. X. (2020). Calcitonin gene-related peptide attenuates angiotensin II-induced ROS- dependent apoptosis in vascular smooth muscle cells by inhibiting the CaMKII/CREB signalling pathway. *Biochemical and Biophysical Research Communications*.

[25] Fan C. D., Sun J. Y., Fu X. T. (2017). Astaxanthin attenuates homocysteine-induced cardiotoxicity in vitro and in vivo by inhibiting mitochondrial dysfunction and oxidative damage. *Frontiers in Physiology*.

[26] Kamerkar S. C., Kraus F., Sharpe A. J., Pucadyil T. J., Ryan M. T. (2018). Dynamin-related protein 1 has membrane constricting and severing abilities sufficient for mitochondrial and peroxisomal fission. *Nature Communications*.

[27] Suzuki M., Jeong S. Y., Karbowski M., Youle R. J., Tjandra N. (2003). The solution structure of human mitochondria fission protein Fis1 reveals a novel TPR-like helix bundle. *Journal of Molecular Biology*.

[28] Ding M., Dong Q., Liu Z. (2017). Inhibition of dynamin-related protein 1 protects against myocardial ischemia-reperfusion injury in diabetic mice. *Cardiovascular Diabetology*.

[29] Xia Y., Chen Z., Chen A. (2017). LCZ696 improves cardiac function via alleviating Drp1-mediated mitochondrial dysfunction in mice with doxorubicin-induced dilated cardiomyopathy. *Journal of Molecular and Cellular Cardiology*.

[30] Ikeda Y., Shirakabe A., Brady C., Zablocki D., Ohishi M., Sadoshima J. (2015). Molecular mechanisms mediating mitochondrial dynamics and mitophagy and their functional roles in the cardiovascular system. *Journal of Molecular and Cellular Cardiology*.

[31] Kubli D. A., Zhang X., Lee Y. (2013). Parkin protein deficiency exacerbates cardiac injury and reduces survival following myocardial infarction. *The Journal of Biological Chemistry*.

[32] Siddall H. K., Yellon D. M., Ong S.-B. (2013). Loss of PINK1 increases the heart's vulnerability to ischemia-reperfusion injury. *PLoS One*.

[33] Chen Y., Dorn G. W. (2013). PINK1-phosphorylated mitofusin 2 is a Parkin receptor for culling damaged mitochondria. *Science*.

[34] Picca A., Lezza A. M. (2015). Regulation of mitochondrial biogenesis through TFAM-mitochondrial DNA interactions: useful insights from aging and calorie restriction studies. *Mitochondrion*.

[35] St-Pierre J., Drori S., Uldry M. (2006). Suppression of reactive oxygen species and neurodegeneration by the PGC-1 transcriptional coactivators. *Cell*.

[36] Visioli F., Artaria C. (2017). Astaxanthin in cardiovascular health and disease: mechanisms of action, therapeutic merits, and knowledge gaps. *Food & Function*.

[37] Pongkan W., Takatori O., Ni Y. (2017). *β*-cryptoxanthin exerts greater cardioprotective effects on cardiac ischemia-reperfusion injury than astaxanthin by attenuating mitochondrial dysfunction in mice. *Molecular Nutrition & Food Research*.

