# Erratum to “Astaxanthin Attenuates Hypertensive Vascular Remodeling by Protecting Vascular Smooth Muscle Cells from Oxidative Stress-Induced Mitochondrial Dysfunction”

**DOI:** 10.1155/2021/9796134

**Published:** 2021-12-11

**Authors:** Yuqiong Chen, Su Li, Yuxuan Guo, Hang Yu, Yandong Bao, Xin Xin, Huimin Yang, Xinzhu Ni, Nan Wu, Dalin Jia

**Affiliations:** ^1^Department of Cardiology, The First Affiliated Hospital of China Medical University, Shenyang, Liaoning, China; ^2^Department of Cardiology, Shanghai Institute of Cardiovascular Diseases, Zhongshan Hospital, Fudan University, Shanghai, China; ^3^The Central Laboratory, The First Affiliated Hospital of China Medical University, Shenyang, Liaoning, China

In the article titled “Astaxanthin Attenuates Hypertensive Vascular Remodeling by Protecting Vascular Smooth Muscle Cells from Oxidative Stress-Induced Mitochondrial Dysfunction” [1], there was an error in Figure 5. A duplicate of the central panel in Figure 5(a) was erroneously uploaded as the final panel in Figure 5(a). The corrected figure is shown below and is listed as Figure 1:

## Figures and Tables

**Figure 1:**
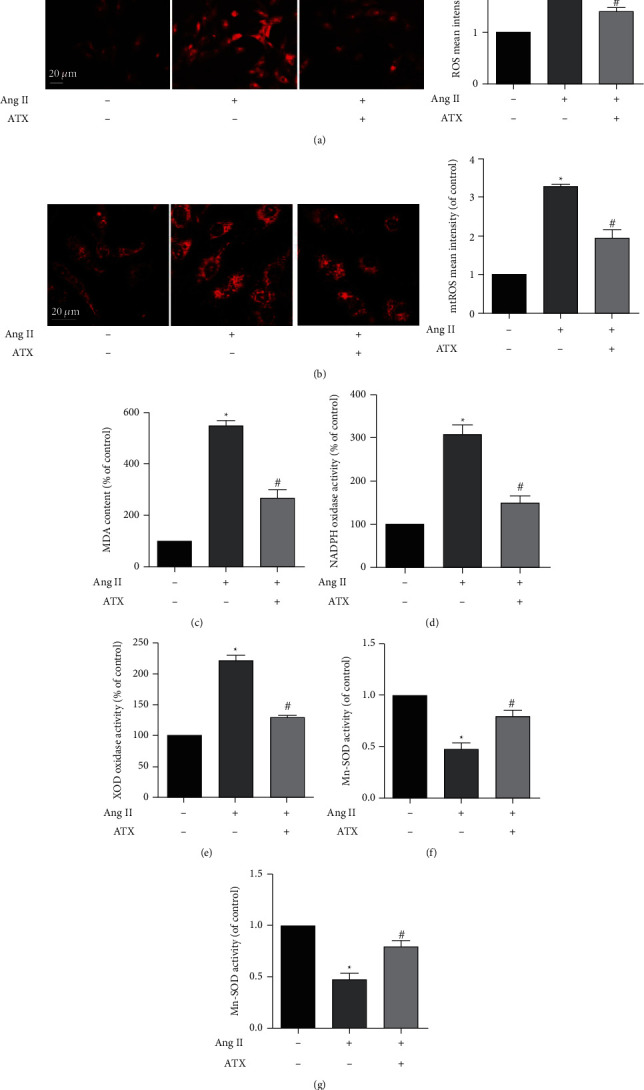
ATX decreases oxidative stress induced by Ang II in VSMCs. (a, b) Cellular ROS and mtROS of VSMCs were imaged by confocal microscopy, and the mean intensity of each group was quantified. Bar = 20 *μ*m. The content of MDA (c) and the enzymatic activities of NADPH oxidase (d), xanthine oxidase (e), SOD (f), and Mn-SOD (g) of VSMCs in each group were analyzed. Values are represented as mean ± SEM of 6 independent experiments. ^∗^*P* < 0.05 vs. untreated controls; ^#^*P* < 0.05 vs. VSMCs injured by Ang II (1 *μ*M).
