# Supplementary material for: Astaxanthin Attenuates Hypertensive Vascular Remodeling by Protecting Vascular Smooth Muscle Cells from Oxidative Stress-Induced Mitochondrial Dysfunction
Source: Oxid Med Cell Longev. 2020 Apr 14;2020:4629189. doi: 10.1155/2020/4629189 (PMC7178508; doi:10.1155/2020/4629189)
Supplement: Supplementary Materials — Table S1: EC50 analyses for phenylephrine and Ca2+-induced vasocontractions. Table S2: maximum response analyses for phenylephrine and Ca2+-induced vasocontractions. [file 4629189.f1.pdf]

Table S1 EC50 analyses for phenylephrine and Ca^{2+}induced vasocontractions.

		WKY	WKY+ATX	SHR	SHR+ATX
	n	8	8	8	8
Phe with E	Mean(log)	-6.14	-6.11	-6.76*	$-6.42^{\#}$
	Sd	0.05	0.04	0.06	0.05
Phe without E	Mean(log)	-7.14	-7.13	-7.34*	$-7.16^{\#}$
	Sd	0.05	0.06	0.07	0.05
Ca^{2+} with E	Mean(log)	-3.82	-3.73	-3.89	-4.02
	Sd	0.95	0.27	0.10	0.81
Ca^{2+}without E	Mean(log)	-3.88	-3.80	-3.88	-3.91
	Sd	0.11	0.12	0.10	0.15

Phe, phenylephrine; E, endothelium. * P<0.05 vs.corresponding WKYs; $^{\#}$ P<0.05 vs. corresponding SHRs.

Table S2 Maximum response analyses for phenylephrine and Ca^{2+}induced vasocontractions.

		WKY	WKY+ATX	SHR	SHR+ATX
	n	8	8	8	8
Phe with E	Mean (%)	97.19	96.79	139.00*	$104.37^{\#}$
	Sd	9.12	12.83	15.27	11.70
Phe without E	Mean (%)	157.73	161.69	207.65*	$176.70^{\#}$
	Sd	15.96	16.81	18.30	13.26
Ca^{2+} with E	Mean (%)	99.83	93.83	147.40*	$117.85^{\#}$
	Sd	18.71	13.54	18.31	18.83
Ca^{2+}without E	Mean (%)	135.46	129.46	182.22*	$154.44^{\#}$
	Sd	13.60	8.97	16.01	18.50

Phe, phenylephrine; E, endothelium. * P<0.05 vs.corresponding WKYs; $^{\#}$ P<0.05 vs. corresponding SHRs.
